# Correlates of Mortal Distress Among Healthcare Staff in Hospitals: A Systematic Review and Meta‐Analysis

**DOI:** 10.1111/jan.70454

**Published:** 2026-03-04

**Authors:** Joy Juan Wang, Jing Ning, Jiu Li Xu, Yong Hao Ng, Ernest Wing‐Tak Chui

**Affiliations:** ^1^ The Nethersole School of Nursing, Faculty of Medicine The Chinese University of Hong Kong Hong Kong, China; ^2^ Department of Social Work and Social Administration, Faculty of Social Sciences The University of Hong Kong Hong Kong, China; ^3^ China Resources Hong Kong, China; ^4^ Department of Social Work, Faculty of Arts and Social Sciences National University of Singapore Singapore; ^5^ University of Western Australia and Perron Institute for Neurological and Translational Science Perth Western Australia Australia

**Keywords:** correlates, death and dying, death anxiety, distress, fear of death, healthcare staff, meta‐analysis, systematic review

## Abstract

**Background:**

Mortal distress encompasses emotional, cognitive, physical and behavioural responses to death and dying among healthcare staff who frequently encounter mortality in hospital settings. Healthcare workers often experience heightened levels of mortal distress due to their regular exposure to patient deaths, which can negatively impact both their personal and professional lives, leading to burnout and high turnover rates.

**Aim:**

To identify and quantitatively synthesise correlates of mortal distress among hospital healthcare staff and examine moderating factors affecting these relationships.

**Design:**

Systematic review and meta‐analysis following PRISMA 2020 guidelines

**Methods:**

Two independent reviewers screened and extracted data from studies published between January 1990 and December 2024 across eight databases (five English: CINAHL, MEDLINE, ProQuest, PubMed, Scopus; three Chinese: Airiti, CNKI, Wanfang). Quality assessment was conducted using the Mixed Methods Appraisal Tool. Meta‐analysis was performed using Comprehensive Meta‐Analysis 3.0.

**Results:**

Analysis of 94 studies identified three factor domains: personal, job‐related and situational. Four job‐related factors demonstrated the strongest correlations with mortal distress: competence in coping with death in healthcare contexts, needs for death‐related or hospice care training, quality of end‐of‐life communication, and working in departments with high patient mortality rates. Four significant moderators influenced correlation strength: publication language, geographic region, study quality, and measurement tools used for assessing mortal distress.

**Conclusions:**

This synthesis provides evidence regarding the magnitude and strength of factors associated with mortal distress among healthcare staff. The identification of main and moderator effects emphasises the critical need for developing culturally sensitive, tailored interventions to help healthcare workers navigate mortality‐related challenges.

**Implications:**

The results can guide healthcare organisations in developing targeted interventions and training programs, inform medical and nursing education curricula by encouraging the inclusion of life and death education, and ultimately enhance staff well‐being while improving the quality of patient and family care, especially in palliative care contexts.

**No Patient or Public Contribution:**

This study did not include patient or public involvement in its design, conduct, or reporting.

**Trial Registration:**

PROSPERO number: CRD42021275460

## 1 Introduction

Mortality is universal, and it affects not only the family and friends of the deceased but also creates a “ripple effect,” evoking distress in healthcare staff who are frequently exposed to death and dying (Hanna et al. 2024). Such exposure to death may lead to heightened levels of distressing reactions, such as fear, anxiety, regret, and guilt (Medina‐Fernández et al. 2023; Alhusamiah and Zeilani 2023). These reactions are collectively referred to as mortal distress.

Built on concepts of fear of death and death anxiety, mortal distress describes the phenomenon of reacting to death and dying, encompassing a range of emotional, cognitive, physical and behavioural responses that individuals may experience when confronted with mortality (Wang 2025). This phenomenon is prevalent across various cultures and represents a normal human reaction to death and dying. However, excessive mortal distress experienced by healthcare staff has been widely shown to result in adverse consequences, including fatigue, burnout, psychological distress, hesitation in communication with dying patients, and high turnover rates (Özkıriş et al. 2011; Delafontaine et al. 2024).

Mortal distress among healthcare staff has gained increasing attention in empirical research in Western countries and Chinese communities (Hu and Yang 2021; Medina‐Fernández et al. 2023; Nia et al. 2016; Zhang et al. 2022). The COVID‐19 pandemic has further intensified interest in this area, resulting in a surge of studies examining mortal distress among healthcare staff (Jazaiery et al. 2022; Kavaklı et al. 2020).

Despite the growing body of literature on mortal distress, empirical findings regarding the influence of factors such as religion, gender, and work experience remain mixed (Black 2007; Chang et al. 2016; Chang and Lin 2023; Luo et al. 2021; Xue et al. 2020). This inconsistency underscores the necessity for a systematic review and meta‐analysis to synthesise existing evidence. Although recent systematic reviews have attempted to address the research and practical gaps related to factors and experiences of mortal distress, their findings have limitations that hinder their applicability.

First, some reviews have been constrained to specific domains, such as psychological or spiritual factors, limiting a comprehensive understanding of factors associated with mortal distress (Letzner 2024; Menzies et al. 2024). A more comprehensive examination of the factors and outcomes of mortal distress is needed to fill this gap, one that considers multiple relevant domains, such as personal experiences, professional factors and contextual factors.

Furthermore, previous systematic reviews about healthcare staff are limited. They focused on specific domain factors (e.g., religiosity and spirituality or medical communication) (Draper et al. 2019; Saeidi and Hamidi 2024) or health crisis (e.g., COVID‐19) (Ghazanfari et al. 2022), or groups (e.g., nurses or physicians only) (Norouzi et al. 2022; Zheng et al. 2016). This impedes a comprehensive understanding of the experiences of all healthcare staff in hospital settings. Moreover, the lack of quantitative synthesis of the findings highlights a critical gap in literature. Therefore, a meta‐analysis specifically focused on the multifarious factors and mortal distress among healthcare staff is warranted. Such a study will enable the quantitative synthesis of existing evidence, estimation of overall effect sizes.

Last but not least, previous reviews have not examined moderators that may critically shape the relationship between the correlates and mortal distress. Potential moderators—such as the measurement tools used to assess death anxiety and fear of death, the language of publication, geographic region of the research, and methodological quality of the studies—may significantly affect these associations. The identification of potential sources of heterogeneity is also crucial.

Therefore, this systematic review aims to address the identified gaps by systematically examining both the influencing and outcome factors of mortal distress among healthcare staff. By including a broad range of healthcare staff, this review seeks to offer comprehensive insights and practical implications for individuals and the healthcare system as a whole. The central research question is: What are the correlates of mortal distress among hospital healthcare staff? Ultimately, the findings from this systematic review will deepen our understanding of mortal distress in healthcare staff and inform the development of interventions and support systems to mitigate their mortal distress and enhance their well‐being.

## 2 Methods

The systematic review was registered with the International Prospective Register of Systematic Reviews (PROSPERO) database (ID number: CRD42021275460). The review is reported following the Preferred Reporting Items for Systematic Reviews and Meta‐Analyzes (PRISMA 2020) guidelines (Page et al. 2021).

### 2.1 Search Strategy

Articles published between January 1990 and December 2024 were searched through eight electronic databases, including five English ones, MEDLINE, Nursing and Allied Health Literature (CINAHL), ProQuest, PubMed, Scopus and three Chinese ones, which are Airiti, Chinese National Knowledge Infrastructure (CNKI), and WanFang. The search terms are developed based on four components, namely (1) Distress, (2) Death, (3) Healthcare, and (4) Professionals. Specifically, the terms utilised were “anxiety OR fear* OR phobia OR distress” AND “death OR dying OR mortal*” AND “health OR healthcare OR medical” AND “staff* OR professional* OR doctor* OR nurs* OR physician* OR social worker* OR therapist*”.

### 2.2 Inclusion and Exclusion Criteria

The following eligibility criteria are applied in this systematic review: (1) fear of death, death anxiety or equivalent concepts of mortal distress are investigated, and related measurement tools are employed for assessment; (2) observational or self‐reported studies are used, which are limited to quantitative or mixed‐methods studies; and (3) participating subjects involve healthcare staff, including but not limited to doctors, nurses, physicians, social workers, therapists, dietitians, psychologists, chiropodists, and pharmacists; (4) healthcare staff working in hospitals or hospice settings; (5) correlates of fear of death, death anxiety or equivalent concepts of mortal distress are reported; and (6) published in English or Chinese languages; and (7) full text is accessible. To supplement, given that hospices share organisational structures with hospitals (Artico et al. 2022; de Graaf et al. 2024) and that hospice care is regarded as a form of hospital care (Lu et al. 2024; Park et al. 2021), we include hospital/hospice in the criteria to ensure a more comprehensive capture of experiences relevant to mortal distress across these care settings. Studies are excluded if they are review papers, study protocols, secondary analysis of an original study, or clinical notes.

To minimise the potential biases and ensure the selection of relevant and high‐quality studies for the systematic review, two reviewers (J.W. and J.X.) independently screened the titles and abstracts based on the specified eligibility criteria after removing duplicates. Full‐text articles of potentially eligible studies were retrieved and assessed for final inclusion. The selection process follows the Preferred Reporting Items for Systematic Reviews and Meta‐Analyses (PRISMA) guidelines, and the number of selected and unqualified articles at each stage was recorded. Disagreements that arose between the two reviewers (J.W. and J.X.) during the selection process were successfully addressed through a process of discussion involving a third researcher (M.S.), as well as consultation with our research team's subject matter expert (A. C.) who possesses professional expertise in both research and practical fields related to thanatology.

### 2.3 Data Extraction and Coding

Two co‐authors (J.W. and J.X.) independently extracted relevant information from the included studies. A pre‐piloted data extraction form was utilised to record the extracted data systematically. This included details such as the title, authors, publication year, sample regions, sample size and structure, sampling and survey methods, research design, measurement tools used for assessing mortal distress and related factors, correlational factors, effect size (Pearson's *r*), or other data that could be converted to Pearson's *r*. Additionally, the authors recorded whether the extracted factors were influencing or outcome factors. Disagreements or discrepancies were resolved through group discussion with another co‐author (J.N.). This rigorous and collaborative data extraction process followed established guidelines, ensuring the accuracy and reliability of the extracted data for the systematic review and meta‐analysis. In addition to the data extraction form, all factors and their corresponding correlational coefficients from each study were exported into a meta‐data spreadsheet in a standardised format for further analysis. In cases where Pearson's *r* was not reported, we followed the guidelines outlined by Lipsey and Wilson (2001) to transform the relevant statistical data into Pearson's *r*. An online calculator was employed to compute the effect size accordingly (Lenhard and Lenhard 2022).

### 2.4 Quality Appraisal

The quality of each article was evaluated using the Mix Methods Appraisal Tool (MMAT) Version 2018 (Hong et al. 2018). This tool includes two overarching questions and five additional questions tailored to the specific research design used in each article. Each question was rated as “Yes,” “No,” or “Cannot tell.” The number of design‐specific criteria met on the MMAT was then used to calculate a percentage indicative of overall quality. This percentage was then categorised as follows: very low (≤ 20%), low (40%), moderate (60%), high (80%), and very high (100%) (Hong et al. 2018).

The assessment tool encompassed various domains, such as the sampling strategy, representativeness of the sample, measurement tool employed, risk of bias related to non‐respondents, and appropriateness of the statistical analysis. A multidisciplinary group of researchers with medicine, social work, and psychology backgrounds is involved in the quality assessment process. The first author (J.W.) and another researcher with a medical background (N.W.) conducted the quality assessment and made records separately. Discrepancies are resolved through consultation with another co‐author (J.N.) and research team discussions. Results of the quality assessment for each study are presented in the Table S1.

### 2.5 Data Analysis and Synthesis

The following criteria were applied to select studies for inclusion in the meta‐analysis: (1) Studies that report the effect size using Pearson correlation coefficient or other types of data (for instance, the mean and standard deviation of the *t*‐test and the regression coefficient) that can be converted to Pearson's *r* were included. Studies that employ a measurement tool to assess mortal distress were included. Studies that did not utilise a measurement tool or any factor with data from fewer than two independent studies were excluded from the meta‐analysis (Valentine et al. 2010).

The meta‐analysis was conducted using Comprehensive Meta‐Analysis (CMA) Version 3 software (Borenstein et al. 2022). As for data synthesis, the correlational coefficients, sample sizes, and factors reported in the included studies were pooled for the meta‐analysis. Pearson's correlation coefficient (*r*) and its corresponding 95% confidence interval were used to estimate effect sizes for the correlations between mortal distress and each factor, employing a random‐effects model. A forest plot was used to visually represent the estimated correlations between the factors and mortal distress. To examine the influence of potential moderators on the relationship between the main factors and mortal distress, a random‐effects model was employed. In line with Sterne et al.'s methodological recommendations, moderator analyses were conducted only for factors represented by data from more than 10 studies, to ensure adequate statistical power and reliable estimates in subgroup analyses (Sterne et al. 2008). To assess the heterogeneity between the studies included, the *I* ^*2*^ index and Cochran's *Q* test were employed. According to Higgins et al. (2003), an *I* ^*2*^ value of 25%, 50%, and 75% could be considered low, moderate, and high levels of heterogeneity. Sensitivity analysis was performed by the “leave‐one‐out” procedure to explore the robustness of the results. The overall effect size was recalculated each time to assess the influence of individual studies on the overall results. A funnel plot was used to assess potential publication bias or small‐study effects. Egger's test was employed to quantify the asymmetry in the funnel plot and determine its statistical significance. Following the recommendation by Sterne et al. (2011), the test for funnel plot asymmetry was conducted only when the number of studies exceeded 10.

## 3 Results

### 3.1 Overview of the Studies

**FIGURE 1:**
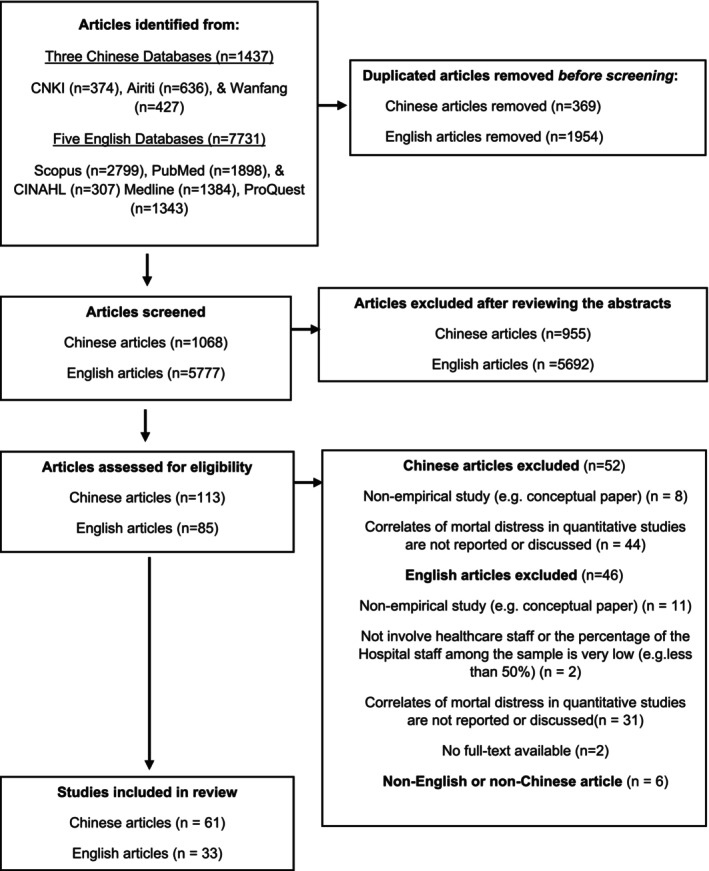
PRISMA diagram‐ process of study selection.

The search for relevant studies occurred in two rounds. The initial round in March 2022 focused on identifying literature published since January 1990 related to the correlates of mortal distress among healthcare staff in hospitals. To incorporate the most recent evidence, a second round was conducted, expanding the search until December 2024. This update aimed to capture any new studies or publications since the initial search. The study selection process is shown in Figure 1 (PRISMA Diagram‐Process of Study Selection).

### 3.2 Studies Characteristics

The majority of the included articles (*n* = 79) were conducted in Asia, followed by Europe (*n* = 10) and North America (*n* = 5). Thirty‐three out of the 94 included articles were published in English, and the rest were published in Chinese (*n* = 61), and 14 were related to the COVID‐19 pandemic.

The reviewed studies predominantly employed a cross‐sectional research design (*n* = 86) and convenience sampling method. The summary of measurement tools used in the included studies is shown in Table 1 below.

**TABLE 1:** Measurement tools used in the included studies.

Measurement tools	Dimensions	Focus	Studies in English	Studies in Chinese
Templer's Death Anxiety Scale (T‐DAS)	Unidimensional	One's own death	(Farhadi et al. 2022; Izadi and Farokhzad 2021; Khajoei et al. 2022; Rahman et al. 2021; Zheng et al. 2016)	(Chen and Yang 2020; He et al. 2019; He et al. 2020; Hu et al. 2015; Huang et al. 2020; Li et al. 2022; Tang et al. 2022; Xue et al. 2020; Yang et al. 2022; Zhang et al. 2022; Zhao et al. 2023; Zhou et al. 2021)
Death Attitude Profile‐Revised (DAP‐R)	Five Dimensions: approach acceptance, fear of death, death avoidance, escape acceptance, and neutral acceptance	One's own death	(Black 2005; Black 2007; Chang and Lin 2023; Dunn et al. 2005; Gama et al. 2012; Gama et al. 2014; Latha et al. 2013; Moudi et al. 2017; Peters et al. 2013)	(Bian 2020; Cao et al. 2019; Chen et al. 2014; Chen and Lin 2004; Cui et al. 2012; Ding and Jin 2020; Du et al. 2020; Xu et al. 2024; Gao et al. 2018; Gou 2020; Guo et al. 2020; Hu and Yang 2021; Huang et al. 2020; Li et al. 2022; Lin et al. 2010; Lin et al. 2022; Liu 2013; Luo 2023; Luo et al. 2019; Luo et al. 2020; Luo et al. 2021; Meng et al. 2014; Qiu et al. 2020; Sheng and Huang 2022; Shi et al. 2019; Tang et al. 2021; Wan et al. 2023; Wang 2017; Wang et al. 2013; Wu and Zhang 2020; Xu et al. 2016; Xu and Lu 2019; Xu et al. 2021; Yang et al. 2023; Yang and Liang 2023; Zeng et al. 2019; Zeng et al. 2022; Zhang, Wen, et al. 2024; Zhang and Li 2020; Zhang and Wu 2018; Zhao, Wang, et al. 2022)
Collett‐Lester Fear of Death Scale (CLFDS)	Four Dimensions: dying of oneself, death of self, dying of others, and death of others	One's own death and others' death	(Asif et al. 2022; Lázaro‐Pérez et al. 2020; Martínez‐López et al. 2021; Medina‐Fernández et al. 2023; Vázquez‐García et al. 2023)	(Chang et al. 2016; Chen and Lin 2004; Chen and Wu 2008; Sun et al. 2005)
Multidimensional Fear of Death Scale (MFODS)	Eight dimensions: fear of dying process, fear of dead, fear of being destroyed, fear of significant others, fear of unknown, fear of conscious death, fear of body after death, fear of premature death,	One's own death	(Roff et al. 2002; Zana et al. 2020)	N/A
Fear of Personal Death Scale (FPDS)	Three dimensions, intrapersonal, interpersonal and transpersonal	One's own death	(Hamama‐Raz et al. 2000)	N/A
Other measurements (e.g., Templer/McMordie Death Anxiety Scale, Thorson‐Powell Death Anxiety Scale Death Anxiety Scale‐Traits)		One's own death	(Bené and Foxall 1991; Çekiç et al. 2022; Kagan 2021; Melo and Oliver 2011; Ratiu et al. 2022; Samson and Shvartzman 2018; Zhao, Liu, and Wang 2022)	(Xu et al. 2024)

*Note:* The specific studies, indexed by study code, are shown in Table S1.

These findings suggest conceptual divergence across different studies, as well as heterogeneity in the measurement tools employed in the existing literature. A summary of the characteristics and the quality assessment score of each included article is presented in Table S1.

### 3.3 Categorization of Factors

This systematic review identified multifarious factors that are associated with healthcare staff's distress evoked by death or dying. Overall, the factors are further categorised into three domains: (1) personal, (2) job, and (3) situation. The categorization of the domains of the factors has undergone discussions with the research team members, including experts with professional expertise in both theoretical and practical fields of thanatology. The primary basis for the categorization is the nature of the correlates of the mortal distress. Factors related to demographics and individual characteristics are classified under the personal domain. Factors associated with the work environment, occupational setting, and professional attributes are grouped into the job‐related domain. Finally, factors on broader contextual or situational elements are categorised as the situational domain. This classification helps us have an overall picture of the current factors that have been investigated and the magnitudes of the correlations between these factors and mortal distress.

The personal domain includes factors such as demographics, prior experiences with death, personality traits associated with a tendency to experience negative emotions, psychological distress, meaning in life, and subjective well‐being (Özkıriş et al. 2011; Hu et al. 2015; Izadi and Farokhzad 2022; Karkhah et al. 2024; Zana et al. 2020). The job‐related domain comprises factors such as working department, exposure to patients' death and dying, burnout, end‐of‐life communication, competence in coping with death, death‐related training needs, and attitudes toward the care of dying individuals (Gama et al. 2012 and Gama et al. 2014; Karkhah et al. 2024, Latha et al. 2013; Peters et al. 2013; Ratiu et al. 2022; Samson and Shvartzman 2018; Zheng et al. 2021; Hamama‐Raz et al. 2000). The situational domain encompasses factors related to the COVID‐19 pandemic (Lázaro‐Pérez 2020; Ratiu et al. 2022; Sarfraz et al. 2025). However, as situational factors did not meet the criteria for inclusion in the meta‐analysis, they were excluded from the quantitative synthesis.

### 3.4 Quantitative Synthesis

**FIGURE 2:**
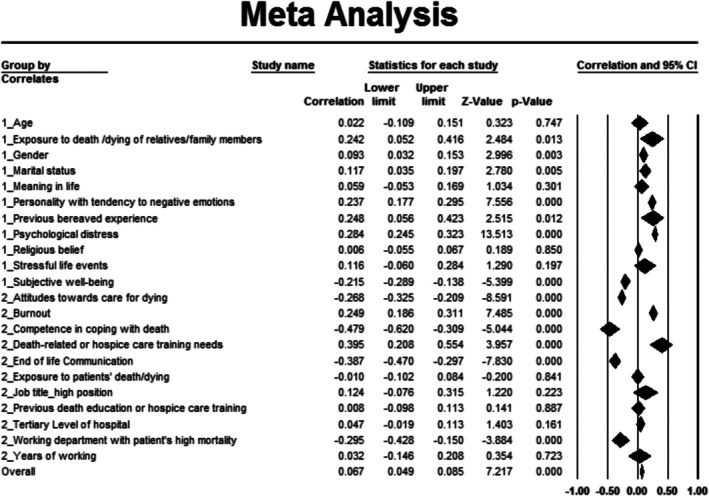
Summary plot of pooled effect sizes of factors. In the first column, “1” denotes personal domain factors and “2” denotes job‐related domain factors. Situational domain factors are not included in the meta‐analysis due to an insufficient number of studies meeting the inclusion criteria. Previous bereaved experience = having previous bereaved experience. Religious belief = having religious belief. Stressful life events = having stressful life events. Death‐related or hospice care training needs = having needs in death‐related training or hospice care training. End‐of‐life communication = quality of end‐of‐life communication. Previous death education or hospice care training = having previous training in death education or hospice care. Working department with patient's high mortality = working in a department with a high patient mortality rate.

Given the heterogeneity of the studies, a random effects model was employed in this meta‐analysis (Higgins et al. 2003). This model accounts for the variability among study results. In Comprehensive Meta‐Analysis (CMA) software, the random‐effects model assumes that the true effects are normally distributed, allowing for the calculation of an average effect size while considering differences between studies (Borenstein et al. 2021). The weights assigned in the random‐effects model are designed to minimise two sources of variance. In comparison to a fixed effects model, the weights in a random‐effects model are more balanced, which helps prevent large studies from dominating the analysis and ensures that smaller studies are not marginalised. The pooled effect size here is equal to the random effect size. Figure 2 is a summary plot that visually presents the pooled effect sizes of the factors analysed in the meta‐analysis. The output of the random effect analysis is shown in Table S2 (the 1st and 2nd columns are the number of studies and the combined effect size for each correlational factor respectively). The coding information for each factor is shown in Table S3. The plot provides an overview of the overall effect sizes and their corresponding confidence intervals for each factor. With reference to the empirical guidelines to interpret the magnitude of the correlation coefficient (Hemphill 2003; Gignac and Szodorai 2016), mild, moderate, and strong correlation coefficients are defined as *r* < 0.2, 0.2 < *r* < 0.3 and *r* > 0.3, respectively. A summary of the relationship between factors and mortal distress is shown in Table 2.

**TABLE 2:** Summary of the relationship between factors and mortal distress.

		Not significant	Statistically significant correlation		
			Mild	Moderate	Strong
Domains	Personal	Age (*k* = 39, *r* = 0.022) Religious belief (*k* = 53, *r* = 0.006) Stressful life event (*k* = 6, *r* = 0.116) Meaning in life (*k* = 12, *r* = 0.059)	Positive Gender (*k* = 40, *r* = 0.093) Marital status (*k* = 25, *r* = 0.117)	Positive: Personality (*k* = 7, *r* = 0.237) Exposure to death or dying of significant others (*k* = 13, *r* = 0.242) Previous bereaved experience (*k* = 7, *r* = 0.248) Psychological distress (*k* = 47, *r* = 0.284) Negative: Subjective well‐being (*k* = 13, *r* = −0.215)	
	Job‐related	Job title with high position (*k* = 10, *r* = 0.124) Exposure to patients' death and dying (*k* = 28, *r* = −0.01) Having previous death education or hospice care training (*k* = 24, *r* = 0.008) Tertiary level of the hospital (*k* = 6, *r* = 0.047) Years of working experience (*k* = 18, *r* = 0.032)		Positive: Burnout (*k* = 33, *r* = 0.249) Negative: Attitudes toward care for dying (*k* = 30, *r* = −0.268)	Positive: Death‐related or hospice care training (*k* = 23, *r* = 0.395). Negative: Competence in coping with death in healthcare context (*k* = 13, *r* = −0.479) Quality of end‐of‐life communication (*k* = 6, *r* = −0.387) Working in a department with a high patient mortality rate (*k* = 6, *r* = −0.295)^a^
	Situational	N/A	N/A	N/A	N/A

*Note:* Situational domain factors are not included in the meta‐analysis due to an insufficient number of studies meeting the inclusion criteria.

^a^ Marginally strong effect size.

As for personal factors, several factors showed a moderate correlation with mortal distress. For example, psychological distress is found to be moderately correlated with mortal distress (*k* = 47, *r* = 0.284). Personality (with the tendency to experience negative emotions) is also found to be moderately correlated with mortal distress (*k* = 7, *r* = 0.237). Previous bereaved experience (*k* = 7, *r* = 0.248) and exposure to death or dying of significant others (*k* = 13, *r* = 0.242) showed moderate positive correlation with mortal distress. Moreover, subjective well‐being is found to have a moderate negative association with mortal distress (*k* = 13, *r* = −0.215). Two factors showed a mild positive correlation with mortal distress, including gender (being female) (*k* = 40, *r* = 0.093) and marital status (married) (*k* = 25, *r* = 0.117).

As for job‐related factors, competence in coping with death (*k* = 13, *r* = −0.479), quality of end‐of‐life communication with patients (*k* = 6, *r* = −0.387), and working in a department with a high patient mortality rate (*k* = 6, *r* = −0.295) are found to have a strong negative correlation with mortal distress. Need for death‐related or hospice care training is found to be a strong positive correlate of mortal distress (*k* = 23, *r* = 0.395). In addition, some other factors with moderate correlation with mortal distress are identified. For example, a moderate negative association between attitudes toward care for dying and mortal distress is found (*k* = 30, *r* = −0.268). It indicates that individuals who experience higher levels of mortal distress tend to have more negative attitudes toward caring for dying patients. Burnout is found to be a moderately positive correlate of mortal distress (*k* = 33, *r* = 0.249).

In addition, some factors are found to be non‐significant with mortal distress. Specifically, age (*k* = 39, *r* = 0.022), religious belief (*k* = 53, *r* = 0.006), stressful life event (*k* = 6, *r* = 0.116), meaning in life (*k* = 12, *r* = 0.059), job title with high position (*k* = 10, *r* = 0.124), exposure to patient's death and dying (*k* = 28, *r* = −0.01), having previous death education or hospice care training (*k* = 24, *r* = 0.008), tertiary level of the hospital (*k* = 6, *r* = 0.047), and years of working experience (*k* = 18, *r* = 0.032) are found to be non‐significant correlates of mortal distress among healthcare staff (*p* > 0.05).

### 3.5 Moderator Analysis

Moderator analyses were conducted to verify if the potential moderators can explain the extra heterogeneity across studies. In this study, several reasons were considered for including the moderators of region, language, quality assessment score (MMAT), and measurement tools for assessing the mortal distress in the analysis. First, experiences of mortal distress vary across cultural contexts, with cultural values and beliefs influencing perceptions in hospital settings (Cao et al. 2019; Martín‐Parrilla et al. 2025). By considering language and regional differences, we aim to capture how these cultural factors affect the relationship between correlates and mortal distress. Additionally, various measurement tools for assessing mortal distress exist (Zuccala et al. 2022), and their variability across studies may impact the associations. Finally, the methodological quality of each study, as measured by the MMAT score, can significantly influence associations, prompting an examination of sources of heterogeneity. Notably, some findings should be interpreted with caution due to the small number of studies in certain subgroups. A summary of moderator analyses is provided in Table S4.

Specifically, the region was classified into four subgroups: 1 = Europe, 2 = North America, 3 = South America, and 4 = Asia. The analysis shows a statistically significant moderating effect in the correlation between mortal distress and four factors, including age (*Q*(2) = 21.597, *p* < 0.001), religious belief (*Q*(1) = 17.059, *p* < 0.001), competence in coping with death (*Q*(1) = 92.050, *p* < 0.001), psychological distress (*Q*(1) = 13.772, *p* < 0.001). The effect size between mortal distress and psychological distress is much stronger in studies from Asia compared to studies from other regions.

The language variable was classified into two subgroups: Chinese and English. The analysis revealed statistically significant moderating effects of language only on the correlations between mortal distress and competence in coping with death (*Q*(1) = 3.991, *p* < 0.05). Specifically, mixed‐effects models indicated a stronger and more statistically significant correlation in studies published in Chinese compared to those published in English.

The quality assessment score (MMAT), ranging from 0 to 5, was treated as a continuous moderator in the analysis. Statistically significant moderating effects of MMAT scores were observed in the correlations between mortal distress and six factors, including marital status (*Q*(2) = 105.619, *p* < 0.001), psychological distress (*Q*(2) = 12.824, *p* < 0.001), attitudes toward death and dying (*Q*(2) = 9.445, *p* < 0.01), death‐related or hospice care training needs (*Q*(1) = 26.944, *p* < 0.001), exposure to death/dying patients (*Q*(2) = 118.506, *p* < 0.001), and burnout (*Q*(2) = 8.499, *p* < 0.05).

Various instruments were used across the included studies to assess mortal distress, including the Death Attitude Profile‐Revised (DAP‐R), Templer's Death Anxiety Scale, Collett‐Lester Fear of Death Scale, Multidimensional Fear of Death Scale, Fear of Personal Death Scale, and others. Moderator analyses revealed that the type of measurement tool significantly moderated the relationships between mortal distress and nine factors: age (*Q*(6) = 42.217, *p* < 0.001), gender (*Q*(6) = 15.393, *p* < 0.01), religious belief (*Q*(6) = 40.721, *p* < 0.001), psychological distress (*Q*(3) = 28.901, *p* < 0.001), subjective well‐being (*Q*(11) = 31.155, *p* < 0.01), competence in coping with death (*Q*(5) = 246.085, *p* < 0.001), exposure to death/dying patients (*Q*(5) = 28.348, *p* < 0.001), years of working experience (*Q*(5) = 11.999, *p* < 0.05), and burnout (*Q*(5) = 71.317, *p* < 0.001).

### 3.6 Sensitivity Analysis

The leave‐one‐out sensitivity method was used for the sensitivity analysis of studies reporting, and no studies were found to significantly impact the results (as shown in Table S5), indicating the stability of the study results.

### 3.7 Publication Bias

The publication bias analysis for each factor (with number of studies > 10) was conducted, and the funnel plots are shown in Figure S1. Most of the factors show symmetry, indicating that biases regarding the association between these factors and mortal distress were not present. However, the factors of marital status, death‐related training needs, hospice care training needs, and competence in coping with death suggested potential asymmetry. It is important to note that, aside from publication bias, there may be other potential causes of funnel plot asymmetry, including the small‐study effect. Specifically: (1) the distribution of effect sizes differs in large studies compared to small studies; (2) methodological quality may vary in smaller studies; and (3) true heterogeneity may exist (Sterne et al. 2011). For example, regarding the association between mortal distress and marital status, there is a clustering of studies at the top and two outliers displaying on the right‐hand side (shown in the Figure S1), indicating a relatively insufficient representation for studies with small sample sizes and smaller studies may report larger effects. Additionally, for the association between mortal distress and death‐related or hospice care training needs and competence in coping with death, the studies are concentrated at the top and right‐hand side of the plot (shown in the Figure S1), with missing studies at the bottom, indicating studies showing significant positive results may be more likely to be published. Subsequent Begg's and Egger's tests were conducted. Egger's regression test indicated statistically significant publication bias in studies examining the association between mortal distress and marital status (*p* = 0.017), needs for death‐related or hospice care training (*p* = 0.002), and competence in coping with death (*p* = 0.009). However, Duval and Tweedie's trim and fill method did not identify any missing studies or adjust the pooled effect size, suggesting that the impact of this bias on the overall estimate may be limited. Nevertheless, the presence of publication bias should be considered when interpreting the relevant results.

## 4 Discussion

This meta‐analysis provides a comprehensive synthesis of factors associated with mortal distress among healthcare staff, drawing on cross‐sectional studies published between January 1990 and December 2024, in English or Chinese. By categorising these correlates into three domains—personal, job‐related, and situational—this review enhances our understanding of the factors associated with mortal distress in hospital settings. Using data from eight electronic databases, we identified 22 factors that were included in the meta‐analysis, offering a broad and nuanced view of the current evidence base.

### 4.1 Main Analysis

Among the personal domain factors, three factors, including previous bereavement experience, exposure to death or dying of significant others, and personality traits (with the tendency to experience negative emotions), were identified as moderate risk factors of mortal distress. This could offer insights for identifying the at‐risk group timely.

Moreover, psychological distress is identified as a positive correlate of mortal distress, and subjective well‐being is found to be a moderate negative correlate of mortal distress. This implies that unaddressed mortal distress may adversely affect healthcare staff's overall well‐being, as their personal and professional lives are closely intertwined (Long et al. 2022; Melo and Oliver 2011). Such distress may not only impact their mental health and quality of life but could also influence their job satisfaction and professional performance. Therefore, interventions that target mitigating healthcare staff's mortal distress are critical.

Four job‐related factors showed the strongest correlations with mortal distress among all factors examined: competence in coping with death, quality of end‐of‐life communication with patients, and the need for death‐related or hospice care training, working in a department with a high patient mortality rate.

Firstly, self‐perceived competence in coping with death was strongly associated with lower levels of mortal distress, likely because it enhances individuals' sense of control and reduces anxiety related to death and dying. This finding aligns with recent studies (Medina‐Fernández et al. 2023; Zheng et al. 2021). Tailored training programs that strengthen personal resources and coping strategies may further enhance healthcare staff's competence and emotional resilience in death‐related situations.

Secondly, the quality of end‐of‐life communication was also a strong negative correlate of mortal distress, consistent with previous research on death anxiety (Nia et al. 2016). Healthcare staff experiencing higher levels of mortal distress often exhibit discomfort and hesitation when initiating discussions about advance directives with dying patients. This suggests that heightened distress may negatively impact both professional performance and the quality of end‐of‐life care, underscoring the need to address their mortal distress to better prepare them to optimise the provision of end‐of‐life care and palliative care.

Thirdly, the need for death‐related or hospice care training arose as a strong positive correlate of mortal distress, highlighting the importance of targeted training to address the unique challenges associated with death and dying (Sheng and Huang 2022; Zhao, Wang, et al. 2022). However, the data provided no evidence for a statistically significant association between healthcare staff's prior training and mortal distress. This suggests that the mere presence of training is insufficient. Potential reasons include the nature of the training (e.g., one‐time sessions versus ongoing programs) and whether the training content is holistic and systematic—addressing not only knowledge and skills but also values and beliefs. Literature indicates that healthcare staff's values and beliefs are crucial in shaping their experience of mortal distress (Dzierżanowski and Kozlowski 2022; Romão et al. 2025; Tu et al. 2022). Therefore, effective training should incorporate reflective practice and debriefing sessions, enabling participants to examine and process their own attitudes toward death and potentially foster positive change. Moreover, cultural appropriateness is a crucial consideration in designing training. Death‐related topics are highly sensitive to cultural and social contexts, and thus, research has begun to focus on incorporating cultural perspectives in death education (Zhang, Lv, et al. 2024). Furthermore, training should address the diverse needs of healthcare staff. For those with trauma‐related experiences, integrating adequate support and trauma‐informed care practices into training can make a significant difference.

Taken together, these factors highlight the importance of developing training programmes that are culturally appropriate, context‐sensitive, holistic, and systematic. Such training should be designed to meet the specific needs of healthcare staff working with death and dying, ultimately helping to more effectively address mortal distress.

Additionally, although the data provided no evidence of a statistically significant effect of exposure to patient death and dying on mortal distress, working in a department with a high patient mortality rate emerged as a marginally strong negative correlate. This finding is consistent with previous studies (Chen and Wu 2008; Lin et al. 2022), suggesting that persistent exposure to death and dying may be a strong indicator of those at greater risk for mortal distress. Organisations should therefore implement timely screening and provide adequate support systems and resources for healthcare staff who are most in need.

Burnout and attitudes toward caring for dying patients were also found to have a moderate correlation with mortal distress, consistent with previous research (Dunn et al. 2005; Melo and Oliver 2011; Zana et al. 2020). These findings suggest that healthcare staff experiencing higher levels of mortal distress are more likely to suffer from burnout and to hold negative attitudes toward caring for dying patients.

### 4.2 Moderator Analysis

The moderator analysis shed light on key sources of heterogeneity in the relationships between various factors and mortal distress. By examining region, language of publication, study quality, and measurement tools, we identified patterns that provide some insights into inconsistencies in the literature. Regional differences significantly moderated associations with age, religious belief, psychological distress, and coping competence, suggesting that cultural and healthcare system variations influence how mortal distress is experienced and reported. Stronger effects in regions such as Asia highlight the need for culturally tailored research and interventions. Language of publication also moderated the link between mortal distress and coping competence, with stronger associations in Chinese‐language studies. This underscores the importance of including non‐English literature to take the cultural context into consideration.

Study quality influenced associations with marital status, psychological distress, and exposure to death‐related experiences, indicating that methodological rigour affects reported relationships and emphasising the need for robust designs. Measurement tools significantly moderate associations with nine factors, reflecting the critical role of instrument selection. The heterogeneity across tools points to the need for validated, multidimensional measures of mortal distress to improve comparability. Overall, both contextual (region, language) and methodological (study quality, measurement) factors contribute substantially to variability in findings. However, caution is warranted due to the limited number of studies in some subgroups.

### 4.3 Limitations and Strengths

Limitations of the study should be acknowledged. First, most of the included studies were cross‐sectional in design, limiting the ability to infer causal relationships between the identified factors and mortal distress. Future longitudinal studies are needed to explore how these associations evolve over time. Second, substantial heterogeneity was observed in region, language of publication, study quality, and measurement tools, which may have contributed to variability in the results. Third, the lack of consensus in the definition of the construct of mortal distress presents problems for the development of a standardised measurement tool. Current definitions often overlook the multifaceted nature of reactions, focusing mainly on emotional dimensions, and thus failing to capture the full complexity of healthcare staff's responses to death and dying (Hoelter 1979; Kelvens 1997; Lehto and Stein 2009). This underscores the need for a more holistic conceptualization of mortal distress in future research. Lastly, this review did not contact study authors to obtain missing effect size data (e.g., Pearson's *r* or convertible statistics) due to time constraints. Future systematic reviews should request unreported data from authors to improve analytical comprehensiveness.

Despite these limitations, this systematic review and meta‐analysis is the first to comprehensively examine the correlates of mortal distress among hospital staff and to quantify the strength of these associations. Additionally, to our knowledge, no previous systematic review has examined the related factors of mortal distress using both English and Chinese databases. Thus, this study broadens the representation on the factors and outcomes associated with mortal distress among healthcare staff. Furthermore, the moderator analyses identified key sources of heterogeneity.

## 5 Conclusion

This systematic review and meta‐analysis provides a comprehensive quantitative synthesis of factors associated with mortal distress among healthcare staff in hospital settings. The moderator analyses reveal influences of cultural context, study quality, and measurement tools, emphasising the importance of methodologically rigorous and culturally sensitive research in this field. These results have implications for healthcare organisations seeking to develop evidence‐based interventions to support staff well‐being. The strong association between mortal distress and modifiable factors such as end‐of‐life care communication skills and coping competence suggests these may be important targets for intervention development. Such efforts may enhance healthcare staff well‐being and potentially improve the quality of care provided to patients and their caregivers.

## Funding

The authors have nothing to report.

## Disclosure

There is a statistician on the author team: Yong Hao Ng.

## Conflicts of Interest

The authors declare no conflicts of interest.

## Supporting information


**Table S1:** Summary table of the characteristics of the reviewed articles
**Table S2:** Random effect analysis.
**Table S3:** Coding information on correlates of mortal distress.
**Table S4:** Summary table of moderator analysis.
**Table S5:** Summary table of sensitivity analysis.
**Figure S1:** Funnel plots for publication bias analysis.

## Data Availability

Data sharing is not applicable to this article, as no new data were created or analyzed in this study.
