# Supplementary material for: Correlates of Mortal Distress Among Healthcare Staff in Hospitals: A Systematic Review and Meta‐Analysis
Source: J Adv Nurs. 2026 Mar 4;82(8):8184–99. doi: 10.1111/jan.70454 (PMC13356434; doi:10.1111/jan.70454)

**Supplementary Materials**

Table S1. *Summary Table of the Characteristics of the Reviewed Articles*

Table S2. *Summary Table of Moderator Analysis*

Table S3*. Summary Table of Sensitivity Analysis*

Table S4. *Funnel Plots for Publication Bias Analysis*

| **Table S1.**  *Summary Table of the Characteristics of the Reviewed Articles* | | | | | | | | | |  |
| --- | --- | --- | --- | --- | --- | --- | --- | --- | --- | --- |
| Author, Year | Study ID | Study Design | Measurement | Country/Region | | Sample Size | Sampling Method | | Correlates / Factors* | MMAT score |
| Lázaro-Pérez et al., 2020 | E03 | cross-sectional | CLFDS | | Spain | 157 | non-probabilistic random sampling | | gender;  the subjective perception of whether the lack of personal protection equipment (PPE); burnout | 4 |
| Samson & Shvartzman,, 2018 | E09 | cross-sectional | Death Anxiety Scale | | Israel | 241 | | cluster sampling | secondary traumatic stress;  burnout | 5 |
| Özkıriş, et al., 2011 | E199 | cross-sectional | T-DAS | | Turkey | 304 | convenience sampling | | gender; frequency of encountering with death & dying; department | 5 |
| Melo & Oliver, 2011 | E18 | mixed-methods | Self-invented death anxiety questionnaire | | Portugal | 150 | convenience sampling | | burnout | 4 |
| Bené and Foxall, 1991 | E32 | cross-sectional | Templer/McMordie Death Anxiety Scale | | USA | 70 | convenience sampling | | frequency of job stress;  severity of job stress | 4 |
| Karkhah et al., 2021 | E33 | cross-sectional | Death Anxiety Scale-Extended (DAS-E) | | Iranian | 325 | census sampling | | age; gender; number of children; surviving parents; years of work experience; mild stressful life events; ward type, shift morning fixed; number of end-of-life patients cared for in the last 3 months; | 5 |
| Roff et al., 2002 | E34 | cross-sectional | MFODS | | Lithuania | 92 | convenience sampling | | religiosity | 5 |
| Martínez‐Lópezet al., 2021 | E35 | cross-sectional | CLFDS | | Spain | 304 | random sampling | | gender;  lack of (PPE); need psychological or psychiatric support; | 5 |
| Ratiu et al., 2022 | E37 | cross-sectional | 1)Mortality salience: a single item; 2) DA as a trait: using the Death Anxiety scale | | Eastern Europe | 253 | convenience sampling | | mortality threat (associated with COVID-19); job satisfaction | 4 |
| Zana et al., 2020 | E221 | cross-sectional | MFODS | | Hungary | 195 | not reported | | perceived stress;  depressive symptoms; vital exhaustion; psychological well-being | 3 |
| Peters et al., 2013 | E49 | cross-sectional | DAP-R | | Australia | 56 | convenience sampling | | work specialty;  coping skills; | 3 |
| Gama et al., 2012 | E71 | cross-sectional | DAP-R | | Portugal | 360 | purposive sampling | | working departments; attachment style; | 5 |
| Latha et al., 2013 | E72 | cross-sectional | 1) DAP-R; 2) T-DAS | | India | 100 | not reported | | age;  religiosity;  meaning in life;  life satisfaction | 3 |
| Rahman, et al., 2020 | E73 | cross-sectional | T-DAS | | Turkey | 382 | non-probability sampling | | spirituality and spiritual care; | 4 |
| Hamama-Raz et al., 2000 | E75 | cross-sectional | Fear of Personal Death (Florian & Kravetz, 1983) | | Israel | 233 | convenience sampling | | age;  gender;  religiosity;  number of years as physicians | 5 |
| Black, 2007 | E78 | cross-sectional | DAP-R | | USA | 135 | purposive sampling | | age; collaboration with others (subdomain of ADCP) | 5 |
| Zheng et al., 2021 | E223 | cross-sectional | C-T-DAS | | China | 298 | convenience sampling | | coping with death; death self-efficacy | 5 |
| Dunn et al., 2005 | E129 | cross-sectional | DAP-R | | USA | 58 | not reported | | attitudes about caring for dying patients | 4 |
| Gama et al., 2014 | E144 | cross-sectional | DAP | | Portugal | 360 | purposive sampling | | burn out | 5 |
| Moudi et al., 2017 | E178 | cross-sectional | T-DAS | | Iran | 142 | convenience sampling | | mental health status | 5 |
| Kagan, 2021 | E189 | cross-sectional | death anxiety: a single-item scale designed by Abdel-Khalek | | Israel | 795 | convenience sampling | | psychological distress | 5 |
| Black, 2005 | E190 | cross-sectional | DAP-R | | USA | 29 | purposive sampling | | advance directives communication | 5 |
| Vázquez-García et al., 2023 | 2 E22 | cross-sectional | CLFDS | | Spain | 230 | Non-probabilistic sampling | | gender;  level of training in emotional self-management;  self-perception of the level of mental health; perception of lack of support by palliative care or mental health professionals; | 4 |
| Khajoei, et al., 2022 | 2 E85 | cross-sectional | T-DAS | | Iran | 230 | census sampling | | humor | 5 |
| Zhao et al., 2022 | 2 E196 | cross-sectional | Death Anxiety Survey Schedule | | mainland China | 705 | purposive and snowball sampling | | hedonic well-being; presence of meaning | 5 |
| Medina-Fernández et al., 2023 | 2 E225 | cross-sectional | CLFDS | | Mexico | 255 | purposive sampling | | coping with death; compassion fatigue; | 5 |
| Asif et al., 2022 | 2 E283 | cross-sectional | CLFDS-Revised | | Pakistan | 135 | convenience sampling | | gender;  experience of death of a patient; experience of death of loved one; | 4 |
| Çekiç et al., 2022 | 2 E350 | cross-sectional | 1）Thorson-Powell Death Anxiety Scale (T-PDAS) 2）Fear of COVID-19 Scale | | Turkey | 250 | not reported | | gender;  department;  changes in physical;  changes in emotional;  changes in thoughts;  changes in family relationships;  changes in relationships with friends;  changes in work relationships;  changes in future plan;  fear of COVID-19; | 4 |
| Izadi & Farokhzad, 2022 | 2 E389 | cross-sectional | T-DAS | | Iran | 156 | convenience sampling | | depression; COVID-19 risk | 5 |
| Farhadi et al., 2022 | 2 E391 | cross-sectional | T-DAS | | Iran | 208 | purposive sampling | | gender; mental health;  religious orientation | 5 |
| Chang & Lin, 2023 | 3E27 | prospective | DAP-R | | Taiwan | 323 | not reported | | age | 5 |
| Duran, et al., 2024 | 3E37 | cross-sectional | DAP-R | | Turkey | 388 | not reported | | anxiety | 4 |
| Sarfraz et al., 2023 | 3E10 | cross-sectional | T-DAS | | Pakistan | 389 | convenience sampling | | vaccine acceptance;  positive religious coping; | 4 |
| Luo et al., 2020 | C611 | cross-sectional | C-DAP-R | | mainland China | 331 | convenience sampling | | education; willingness for death education course;  hospital level; marital status;   openness discussing death topics | 5 |
| Zhang and Li, 2020 | C477 | cross-sectional | C-DAP-R | | mainland China | 148 | convenience sampling | | attitudes towards care of the dying | 5 |
| He et al., 2019 | C344 | cross-sectional | C-T-DAS | | mainland China | 165 | convenience sampling | | attitudes towards care of the dying; empathy; | 5 |
| Xue, 2020 | C256 | cross-sectional | C-T-DAS | | mainland China | 409 | not reported | | age;  gender;  education;  experience of participating in the dying of relatives; systematic training ; | 5 |
| Lin et al., 2022 | C560 | cross-sectional | C-DAP-R | | mainland China | 803 | convenience sampling | | marital status;  previous death education;  work department | 5 |
| Zeng et al., 2019 | C503 | cross-sectional | C-DAP-R | | mainland China | 1021 | convenience sampling or random sampling | | gender;  profession;  years of working experience;  having relevant training; relationship with patients;  marital status;  level of hospital | 5 |
| Zeng et a., 2022 | C509 | cross-sectional | C-DAP-R | | mainland China | 1014 | convenience sampling | | burnout | 5 |
| Tang et al., 2021 | C420 | cross-sectional | C-DAP-R | | mainland China | 568 | convenience sampling | | attitudes towards care of dying | 5 |
| Xu et al., 2021 | C492 | cross-sectional | C-DAP-R | | mainland China | 803 | convenience sampling | | attitudes towards care of dying | 5 |
| He et al., 2020 | C73 | cross-sectional | C-T-DAS | | mainland China | 165 | convenience sampling | | attitudes towards care of dying; empathy; | 5 |
| Cui et al., 2012 | C448 | cross-sectional | C-DAP-R | | mainland China | 108 | convenience sampling | | subjective well-being | 5 |
| Chang et al., 2016 | C973 | cross-sectional | C-CLFDS | | Taiwan | 30 | convenience sampling | | age | 5 |
| Chen et al., 2014 | C702 | cross-sectional | C-DAP-R | | mainland China | 157 | convenience sampling | | no significant finding was reported | 5 |
| Li et al., 2020 | C97 | cross-sectional | C-DAP-R | | mainland China | 206 | convenience sampling | | coping with death | 5 |
| Bian et al., 2020 | C16 | cross-sectional | C-DAP-R | | mainland China | 208 | convenience sampling | | having end-of-life care training | 5 |
| Luo et al., 2019 | C608 | cross-sectional | C-DAP-R | | mainland China | 420 | convenience sampling | | attitudes towards care of dying | 5 |
| Chen and Yang et al., 2020 | C689 | cross-sectional | C-T-DAS | | mainland China | 814 | convenience sampling | | having knowledge in death education; meaning in life; | 5 |
| Xu et al., 2016 | C486 | cross-sectional | C-DAP-R | | mainland China | 226 | convenience sampling | | age;  years of working experience;  religious belief; | 5 |
| Luo et al., 2021 | C609 | cross-sectional | C-DAP-R | | mainland China | 182 | convenience sampling | | death education demand; years of working experience in palliative care;  number of dying patients cared for within one month;  the frequency of death education and training | 5 |
| Sun et al., 2005 | C805 | quasi-experimental | C-CLFDS | | Taiwan | 99 | convenience sampling | | age | 5 |
| Du et al., 2020 | C547 | cross-sectional | C-DAP-R | | mainland China | 575 | convenience sampling | | attitudes towards care of dying | 5 |
| Chen and Lin, 2004 | C1150 | quasi-experimental | 1) C-CLFDS 2) C-DAP-R | | Taiwan | 111 | not reported | | age;  attitudes towards discussing death; years of working experience; | 5 |
| Qiu et al., 2020 | C669 | cross-sectional | C-DAP-R | | mainland China | 57 | random sampling | | attitudes towards care of dying | 3 |
| Xu et al., 2019 | C487 | cross-sectional | C-DAP-R | | mainland China | 251 | convenience sampling | | work engagement | 5 |
| Ding and Jin, 2020 | C331 | cross-sectional | C-DAP-R | | mainland China | 153 | convenience sampling | | attitudes towards care of dying | 4 |
| Huang et al., 2019 | C732 | cross-sectional | C-T-DAS | | mainland China | 409 | convenience sampling | | age;  gender;  previous encounter with patients or family members' death;  previous experience in death education or EOL;  emotional intelligence; | 5 |
| Meng et al., 2014 | C439 | cross-sectional | C-DAP-R | | mainland China | 148 | convenience sampling | | attitudes towards care of dying | 5 |
| Gao et al., 2018 | C718 | cross-sectional | C-DAP-R | | mainland China | 464 | convenience sampling | | education;  having death education training;  meaning in life; | 5 |
| Wu & Zhang, 2020 | C400 | cross-sectional | C-DAP-R | | mainland China | 317 | cluster sampling | | attitudes towards care of dying | 5 |
| Zhao et al., 2022 | C664 | cross-sectional | C-DAP-R | | mainland China | 387 | convenience sampling | | death education demands | 5 |
| Zhang and Wu, 2018 | C284 | cross-sectional | C-DAP-R | | mainland China | 656 | convenience sampling | | attitudes towards care of the dying | 5 |
| Wang, 2017 | C578 | cross-sectional | C-DAP-R | | mainland China | 60 | not reported | | age; marital status; years of working experience;  education;  job title;  previous training on EoL care | 3 |
| Xue, et al., 2020 | C642 | cross-sectional | C-T-DAS | | mainland China | 145 | not reported | | years of working experience | 4 |
| Hu and Yang, 2021 | C81 | cross-sectional | C-DAP-R | | mainland China | 794 | convenience sampling | | marital status;  religious belief;  education;  death education;  having experience in emergency rescue; | 5 |
| Zhang et al., 2022 | C290 | cross-sectional | C-T-DAS | | mainland China | 237 | convenience sampling | | psychological resilience;  active-constructive response; | 5 |
| Shi et al., 2019 | C385 | cross-sectional | C-DAP-R | | mainland China | 132 | cluster sampling | | attitudes towards end-of-life care | 5 |
| Zhou et al., 2021 | C409 | cross-sectional | C-T-DAS | | mainland China | 382 | convenience sampling | | death education;  life-threatening illness;  experience loss of significant others within 5 years;  empathy | 5 |
| Gou et al. 2020 | C376 | cross-sectional | C-DAP-R | | mainland China | 75 | not reported | | gender;  years of working experience;  religious belief;  previous death education | 5 |
| Wang et al., 2013 | C576 | cross-sectional | C-DAP-R | | mainland China | 318 | convenience sampling | | years of working experience;  job title;  attitudes towards hospice care | 5 |
| Hu et al., 2015 | C618 | cross-sectional | C-T-DAS | | mainland China | 387 | convenience sampling | | Education;  Religiosity;  having death education; participation in relatives and friends' end-of-life disposal | 5 |
| Guo et al., 2020 | C674 | cross-sectional | C-DAP-R | | mainland China | 163 | convenience sampling | | Education;  loss of relatives in the past one year;  avoidance in talking about death;  openness in discussing death with family members;  attitudes toward caring of the dying | 5 |
| Cao et al., 2019 | C500 | mixed-methods | C-DAP-R | | mainland China | 56 | not reported | | age;  job title | 4 |
| Huang et al.,2020 | C729 | cross-sectional | C-DAP-R | | mainland China | 528 | convenience sampling | | meaning in life | 5 |
| Chen and Wu, 2008 | C1151 | cross-sectional | C-CLFDS | | Taiwan | 184 | purposive sampling | | years of working experience; religiosity; physical health; having received palliative care training; openness to discussing death topics | 4 |
| Lin et al., 2010 | C891 | cross-sectional | C-T-DAS | | Taiwan | 276 | not specified | | age; coping style | 5 |
| Tang et al., 2022 | C414 | cross-sectional | C-T-DAS | | mainland China | 230 | convenience sampling | | emotional intelligence | 5 |
| Liu, 2013 | C370 | cross-sectional | C-DAP-R | | mainland China | 108 | convenience sampling | | gender; burn out | 5 |
| Yang et al., 2022 | 2C35 | cross-sectional | C-T-DAS | | Mainland China | 702 | convenience Sampling | | age;  years of working experience;  marital status;  education;  job title;  religious belief;  employment mode; working department; having experience in emergency rescue; receiving death education courses | 5 |
| Wan et al., 2023 | 2C2 | cross-sectional | C-DAP-R | | Mainland China | 366 | convenience Sampling | | professional grief | 5 |
| Li et al., 2022 | 2C33 | cross-sectional | C-DAP-R | | Mainland China | 198 | convenience Sampling | | hospice care education needs | 5 |
| Yang et al., 2023 | 2C40 | cross-sectional | C-DAP-R | | Mainland China | 628 | cluster random sampling | | death education needs | 5 |
| Luo, 2023 | 2C52 | cross-sectional | C-DAP-R | | Mainland China | 192 | convenience sampling | | education background; years of working experience;  previous training | 5 |
| Zhao et al., 2023 | 2C57 | cross-sectional | C-T-DAS | | Mainland China | 441 | convenience sampling | | self-esteem;  competence in coping with death;  frequency of participation in death education | 5 |
| Sheng and Huang, 2022 | 2C50 | cross-sectional | C-DAP-R | | Mainland China | 200 | cluster sampling method | | hospice care education needs | 4 |
| Yang and Liang, 2023 | 2C39 | cross-sectional | C-DAP-R | | Mainland China | 652 | not specified | | ethnicity;  religious belief; attending funerals | 5 |
| Yuan et al., 2023 | 3C21 | cross-sectional | C-DAP-R | | Mainland China | 429 | convenience sampling | | competence for hospice care | 5 |
| Xu et al., 2024 | 3C22 | cross-sectional | ICU Nurses Professional Death Avoidance Scale | | Mainland China | 462 | convenience sampling | | age; having death education; attitudes towards ICU care; exposure to death of significant others; exposure to death/dying of patients | 5 |
| Wang et al., 2024 | 3C17 | cross-sectional | C-DAP-R | | Mainland China | 163 | convenience sampling | | age;  gender;  years of working experience;  marital status;  having death education;  experience of caring dying family member; | 5 |
| Zhang et al., 2024 | 3C8 | cross-sectional | C-DAP-R | | Mainland China | 987 | convenience sampling | | coping with death | 5 |
| Li et al., 2024 | 3C15 | cross-sectional | C-T-DAS | | Mainland China | 186 | convenience sampling | | age;  job title;  years of working experience;  exposure to patient’s dying;  having death-related education; burnout; end-of-life stress | 5 |
| Fu et al., 2024 | 3C1 | cross-sectional | C-DAP-R | | Mainland China | 211 | convenience sampling | | death self-efficacy | 5 |

*Note.* * indicates significant findings regarding the correlates or factors of mortal distress are listed in the column. C-T-DAS = Chinese Version of Templer’s Death
Anxiety Scale. C-DAP-R = Chinese Version of Death Attitude Profile-Revised. C-CLFDS = Chinese Version of The Collet-Lester-Fear of Death Scale T-DAS = Templer’s Death Anxiety Scale, DAP-R = Death Attitude Profile-Revised. CLFDS = The Collet-Lester Fear of Death Scale. MFODS = The Multidimensional Fear of Death Scale

**Table S2.**

*Summary Table of Moderator Analysis*

|  |  | **Moderators** | | | |
| --- | --- | --- | --- | --- | --- |
| **Factors** | | **Region** | **Language** | **MMAT Score** | **Mortal Distress Measurement Tool** |
|  | Age | **Q(2)=21.597***** | Q(1)=0.005 | Q(2)=2.062 | **Q(6)=42.217***** |
|  | Gender | Q(1)=1.914 | Q(1)=1.461 | Q(1)=2.088 | **Q(6)=15.393*** |
|  | Marital status | Q(1)=0.584 | Q(1)=0.127 | **Q(2)=105.619***** | Q(4)=6.854 |
|  | Religious belief | **Q(1)=17.059***** | Q(1)=0.184 | Q(2)=3.961 | **Q(6)=40.721***** |
|  | Exposure to death /dying of relatives or family members | / | / | Q(1)=0.377 | Q(3)=2.875 |
|  | Psychological distress | **Q(1)=13.772***** | / | **Q(2)=12.824**** | **Q(3)=28.901***** |
|  | Attitudes towards care for dying | Q(1)=0.928 | Q(1)=0.928 | **Q(2)=9.445**** | Q(3)=0.461 |
|  | Death-related or hospice care training needs | / | / | **Q(1)=26.944***** | Q(3)=0.688 |
|  | Exposure to death/dying of patients | Q(1)=0.037 | Q(1)=0.158 | **Q(2)=118.506***** | **Q(5)=28.348***** |
|  | Having previous death education or hospice care training | Q(1)=0.763 | Q(1)=0.763 | Q(1)=1.315 | Q(4)=2.326 |
|  | Years of working experience | Q(1)=0.050 | Q(1)=1.110 | Q(1)=0.001 | **Q(5)=11.999*** |
|  | Burnout | Q(1)=1.087 | Q(1)=1.446 | **Q(2)=8.499*** | **Q(5)=71.317***** |
|  | Subjective well-being | Q(1)=2.582 | Q(1)=0.420 | Q(1)=1.605 | **Q(11)=31.155**** |
|  | Meaning in life | / | Q(1)=0.349 | Q(1)=0.354 | Q(3)=1.222 |
|  | Competence in coping with death | **Q(1)=92.050***** | **Q(1)=3.991*** | **/** | **Q(5)=246.085***** |

***Note.*** Only factors with data from more than 10 studies were included in the moderator analysis.

**Table S3.**

*Summary Table of Sensitivity Analysis*

| **Mixed** **effects** **analysis** |  | **Effect** **size** **and** **95%interval** | | |  |  | **Heterogeneity** | | | **Sensitivity** **analysis** |
| --- | --- | --- | --- | --- | --- | --- | --- | --- | --- | --- |
|  | k | correlation | lower limit | upper limit | Z-value | p-value | Q-value | df(Q) | p-value | correlation range with study removed |
| 1_Age | 39 | 0.022 | -0.109 | 0.151 | 0.323 | 0.747 |  |  |  | -0.01-0.033 |
| 1_Exposure to | 13 | 0.242 | 0.052 | 0.416 | 2.484 | 0.013 |  |  |  | 0.177-0.265 |
| 1_Gender | 40 | 0.093 | 0.032 | 0.153 | 2.996 | 0.003 |  |  |  | 0.073-0.105 |
| 1_Marital status | 25 | 0.117 | 0.035 | 0.197 | 2.780 | 0.005 |  |  |  | 0.066-0.128 |
| 1_Meaning in | 12 | 0.059 | -0.053 | 0.169 | 1.034 | 0.301 |  |  |  | 0.040-0.098 |
| 1_Personality | 7 | 0.237 | 0.177 | 0.295 | 7.556 | 0.000 |  |  |  | / |
| 1_Previous | 7 | 0.248 | 0.056 | 0.423 | 2.515 | 0.012 |  |  |  | / |
| 1_Psychologic | 47 | 0.284 | 0.245 | 0.323 | 13.513 | 0.000 |  |  |  | 0.279-0.291 |
| 1_Religious | 53 | 0.006 | -0.055 | 0.067 | 0.189 | 0.850 |  |  |  | -0.016-0.015 |
| 1_Stressful life | 6 | 0.116 | -0.060 | 0.284 | 1.290 | 0.197 |  |  |  | / |
| 1_Subjective | 12 | -0.215 | -0.289 | -0.138 | 5.399 | 0.000 |  |  |  | -0.228--0.186 |
| 2_Attitudes | 30 | -0.268 | -0.325 | 0.209 | 8.591 | 0.000 |  |  |  | -0.282--0.260 |
| 2_Burnout | 33 | 0.249 | 0.186 | 0.311 | 7.485 | 0.000 |  |  |  | 0.236-0.258 |
| 2_Competence | 13 | -0.479 | -0.620 | -0.309 | -5.044 | 0.000 |  |  |  | -0.537--0.449 |
| 2_Death-relate | 23 | 0.395 | 0.208 | 0.554 | 3.957 | 0.000 |  |  |  | 0.362-0.422 |
| 2_End of life | 6 | -0.387 | -0.470 | 0.297 | -7.830 | 0.000 |  |  |  | / |
| 2_Exposure to | 28 | -0.010 | -0.102 | 0.084 | 0.200 | 0.841 |  |  |  | -0.047-0.023 |
| 2_Job title_high | 10 | 0.124 | -0.076 | 0.315 | 1.220 | 0.223 |  |  |  | / |
| 2_Previous | 24 | 0.008 | -0.098 | 0.113 | 0.141 | 0.887 |  |  |  | -0.036-0.029 |
| 2_Tertiary | 6 | 0.047 | -0.019 | 0.113 | 1.403 | 0.161 |  |  |  | / |
| 2_Working | 6 | -0.295 | -0.428 | 0.150 | -3.884 | 0.000 |  |  |  | / |
| 2_Years of | 18 | 0.032 | -0.146 | 0.208 | 0.354 | 0.723 |  |  |  | -0.008-0.052 |
| Total between |  |  |  |  |  |  | 499.822 | 21 | 0.000 |  |
| Overall | 458 | 0.067 | 0.049 | 0.085 | 7.217 | 0.000 |  |  |  |  |

**Table S4.**

*Funnel Plots for Publication Bias Analysis*

1_Age

1_ Gender


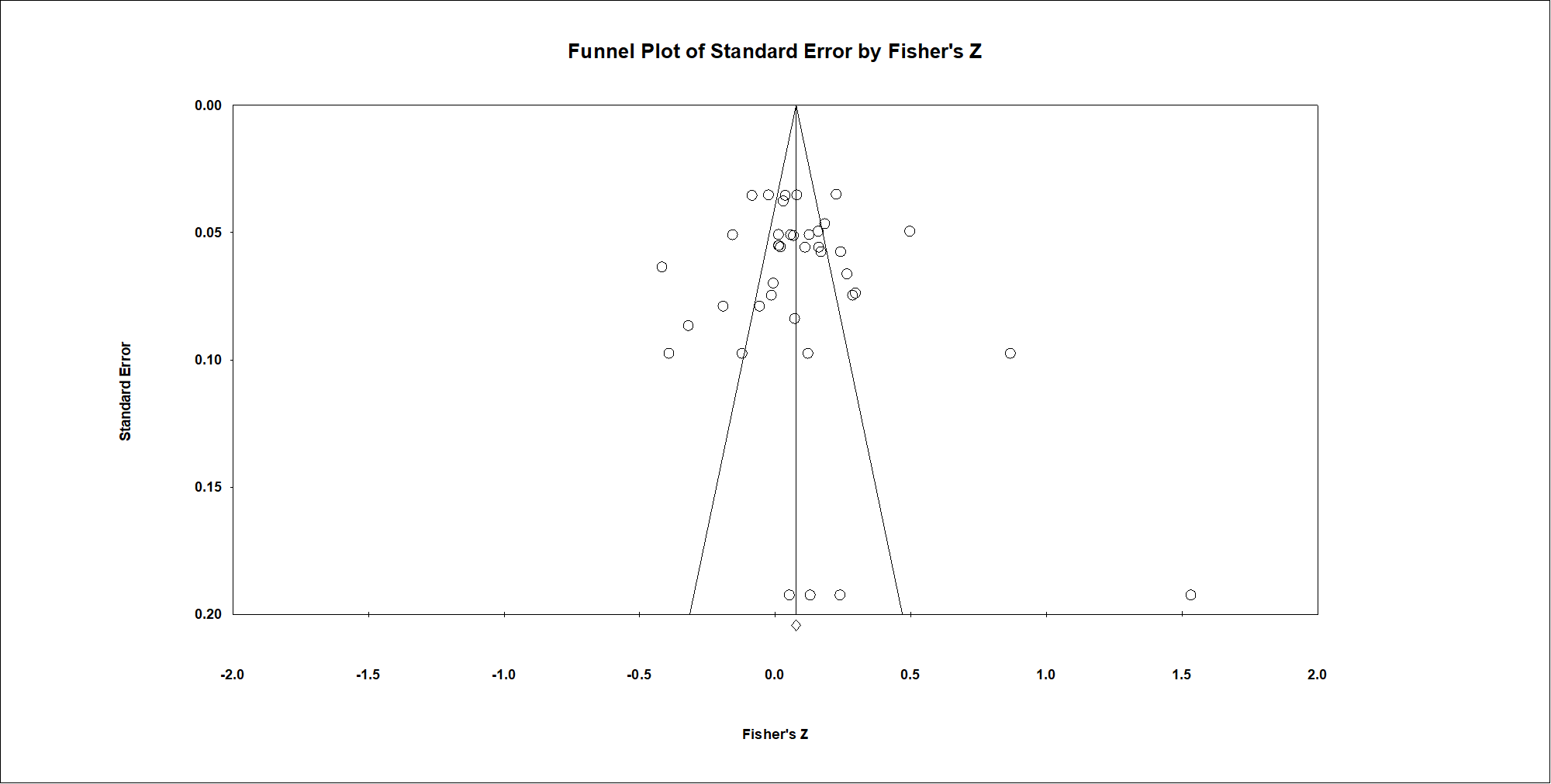


1_Marital Status

1_Religious belief

1_Exposure to death /dying of relatives or family members

1_Psychological distress

1_Subjective well-being

1_Meaning in life

2_Attitudes towards care for dying

2_Death-related or hospice care training needs


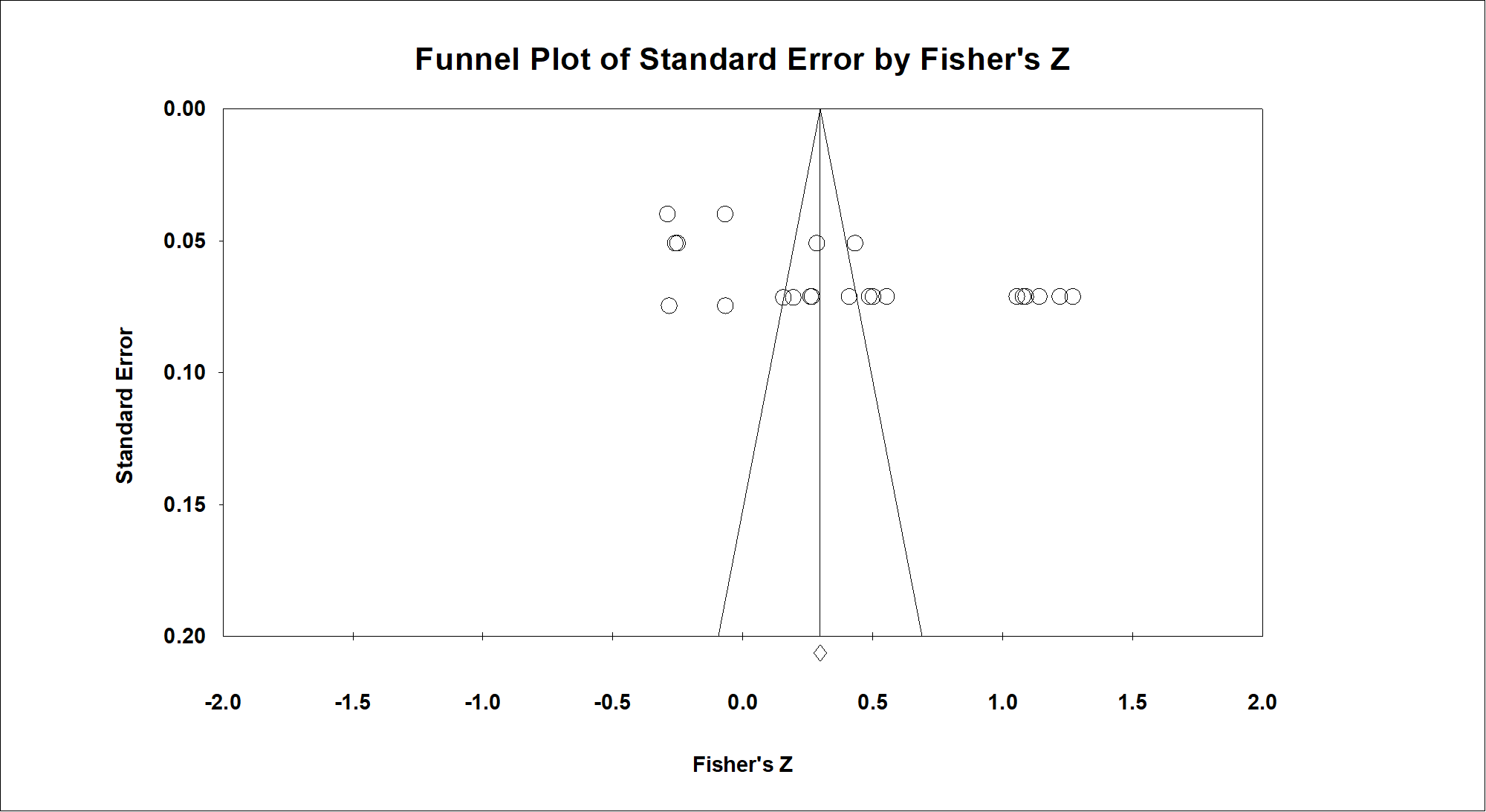


2_Burnout

2_Competence in coping with death


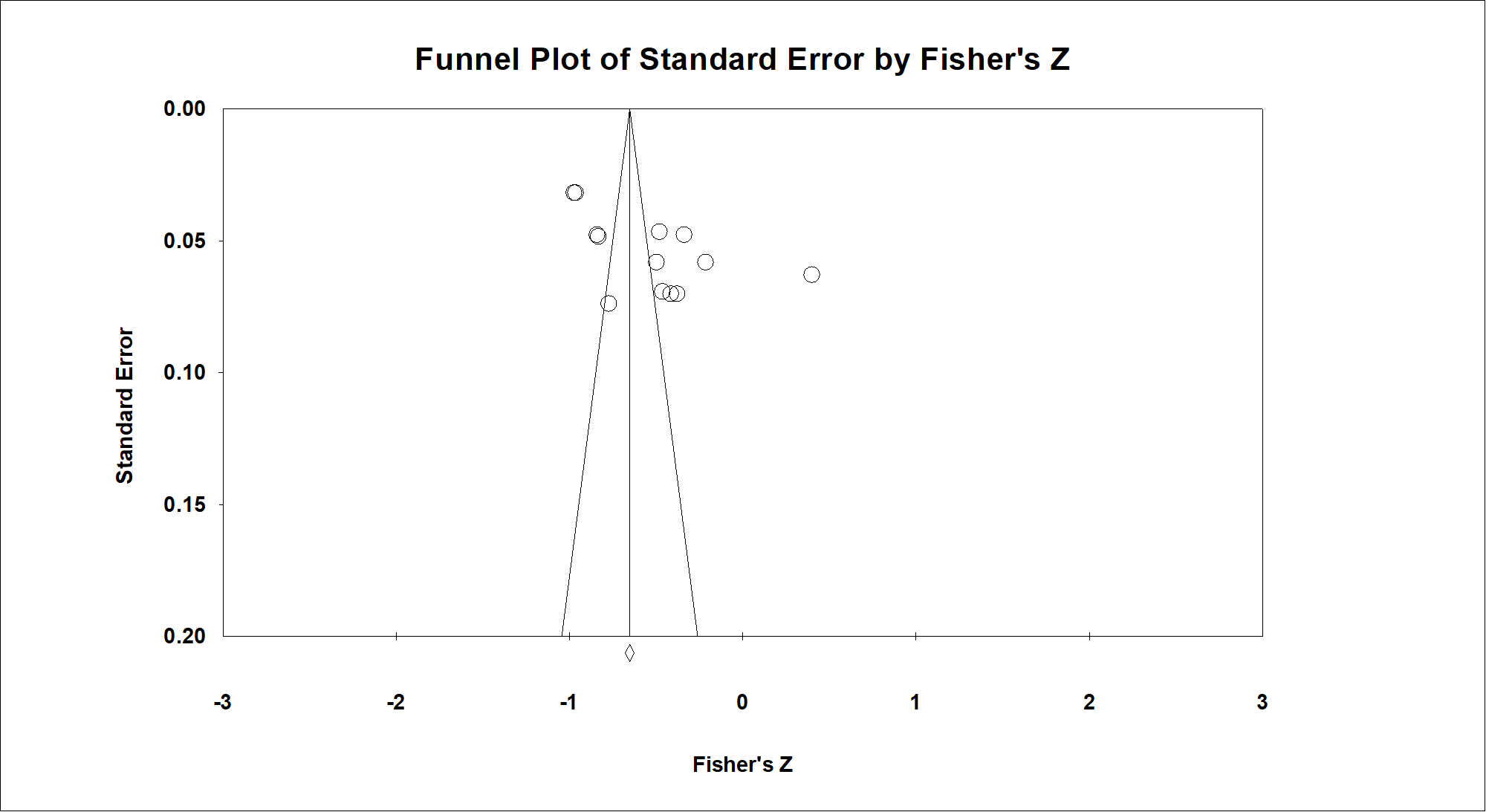


2_Previous death education or hospice care training


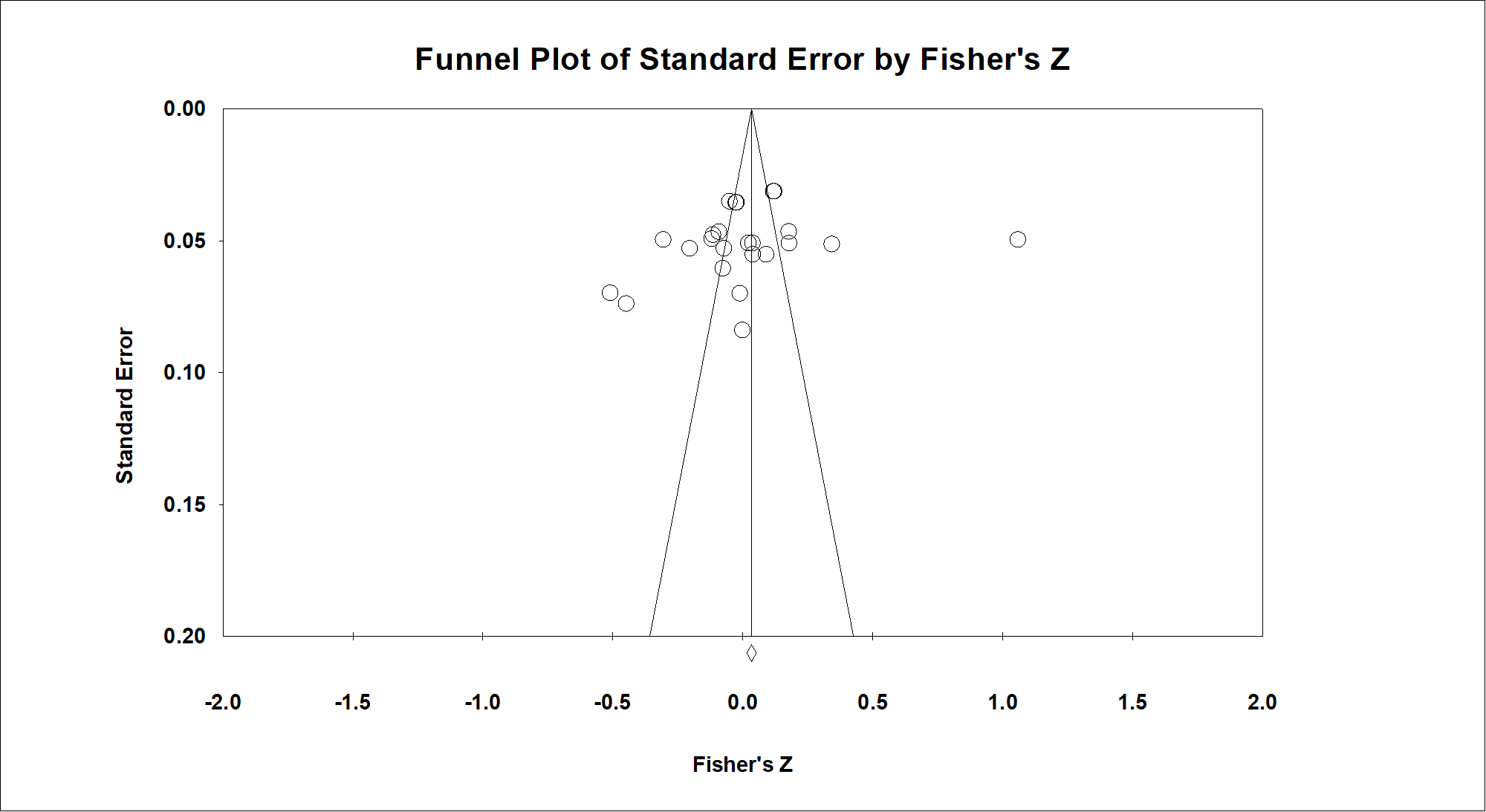


2_Years of working experience


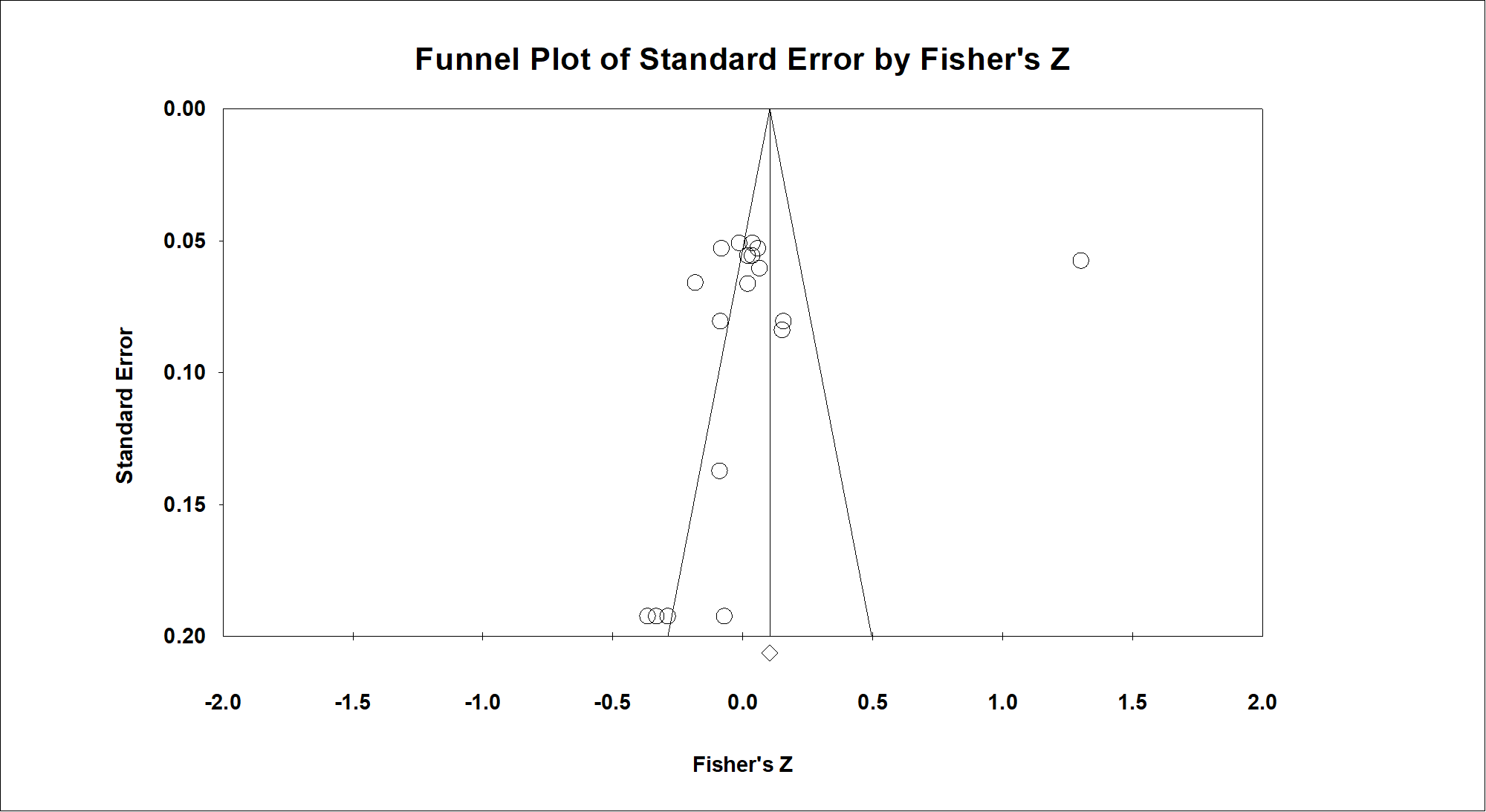


2_ Exposure to death/dying of patients


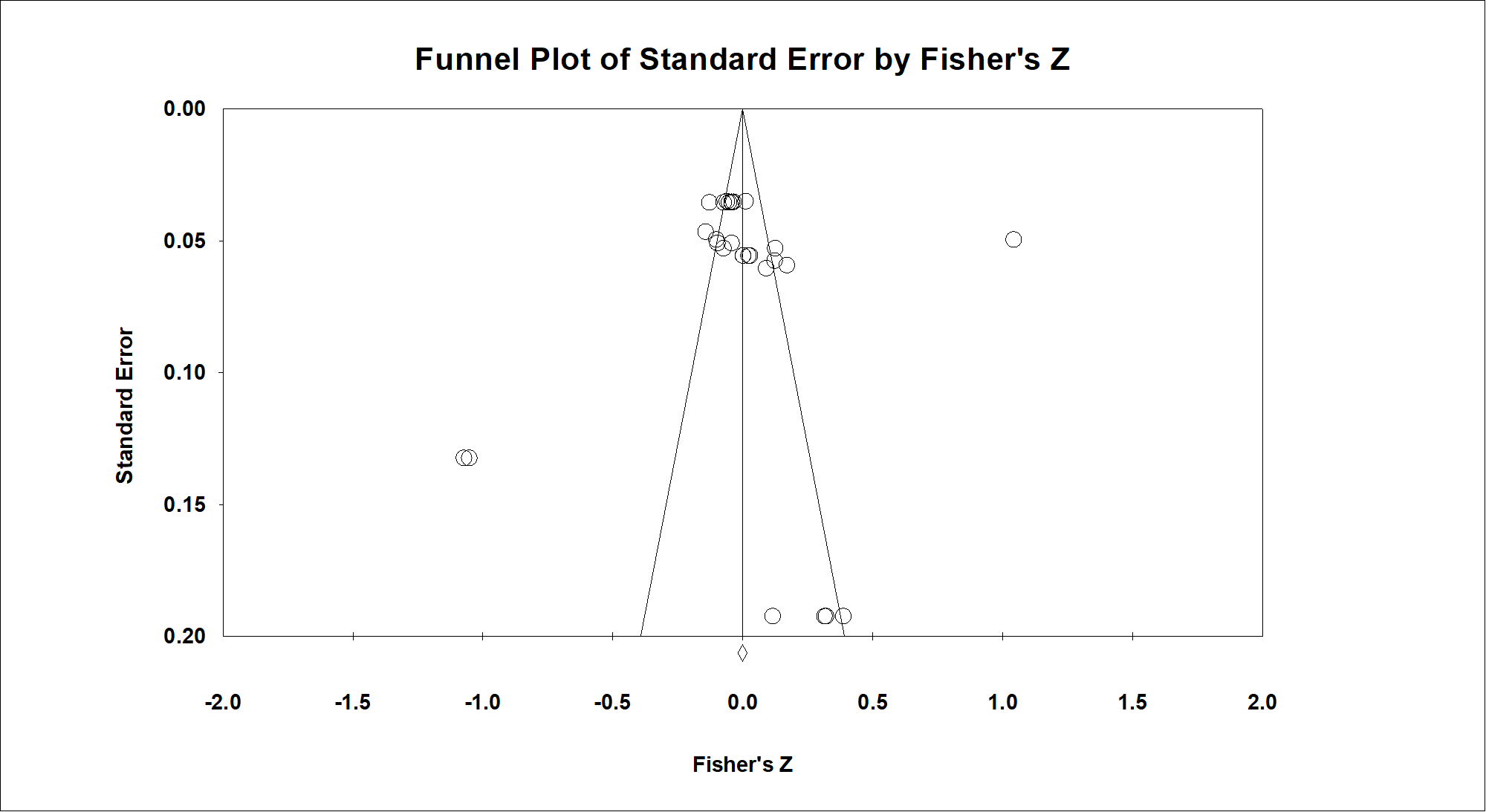

Supplement: Supplementary file 1 — Table S1: Summary table of the characteristics of the reviewed articles Table S2: Random effect analysis. Table S3: Coding information on correlates of mortal distress. Table S4: Summary table of moderator analysis. Table S5: Summary table of sensitivity analysis. Figure S1: Funnel plots for publication bias analysis. [file JAN-82-8184-s001.docx]
